# Pathophysiological mechanisms of root resorption after dental trauma: a systematic scoping review

**DOI:** 10.1186/s12903-021-01510-6

**Published:** 2021-03-26

**Authors:** Kerstin M. Galler, Eva-Maria Grätz, Matthias Widbiller, Wolfgang Buchalla, Helge Knüttel

**Affiliations:** 1 Department of Conservative Dentistry and Periodontology, University Hospital Regensburg, Franz-Josef-Strauß Allee 11, 93053 Regensburg, Germany; 2 University Library, University of Regensburg, Regensburg, Germany

**Keywords:** Root resorption, Tooth resorption, Osteoclast, Receptor activator of nuclear factor-kappa B, RANK ligand, Osteoprotegerin

## Abstract

**Background:**

The objective of this scoping review was to systematically explore the current knowledge of cellular and molecular processes that drive and control trauma-associated root resorption, to identify research gaps and to provide a basis for improved prevention and therapy.

**Methods:**

Four major bibliographic databases were searched according to the research question up to February 2021 and supplemented manually. Reports on physiologic, histologic, anatomic and clinical aspects of root resorption following dental trauma were included. Duplicates were removed, the collected material was screened by title/abstract and assessed for eligibility based on the full text. Relevant aspects were extracted, organized and summarized.

**Results:**

846 papers were identified as relevant for a qualitative summary. Consideration of pathophysiological mechanisms concerning trauma-related root resorption in the literature is sparse. Whereas some forms of resorption have been explored thoroughly, the etiology of others, particularly invasive cervical resorption, is still under debate, resulting in inadequate diagnostics and heterogeneous clinical recommendations. Effective therapies for progressive replacement resorptions have not been established. Whereas the discovery of the RANKL/RANK/OPG system is essential to our understanding of resorptive processes, many questions regarding the functional regulation of osteo-/odontoclasts remain unanswered.

**Conclusions:**

This scoping review provides an overview of existing evidence, but also identifies knowledge gaps that need to be addressed by continued laboratory and clinical research.

**Supplementary Information:**

The online version contains supplementary material available at 10.1186/s12903-021-01510-6.

## Background

Dental trauma presents in countless individual forms and characteristics. The broad spectrum of injury patterns is a consequence of the multiple possible combinations of damage to gingiva, dental hard tissues, pulp, periodontium and alveolar bone [1]. This complexity contributes to an increased incidence of long-term consequences, where combined injuries may cause late effects in 65% of all cases [2–4]. Dental trauma is problematic especially in children, where tooth development and growth of the jaw bones are incomplete, and low compliance may complicate adequate therapies [5]. Not all oral tissues contribute equally to late effects: whereas gingiva and bone heal within days to weeks, damage to dental pulp and periodontal tissues are determinants for poor long-term prognosis of traumatized teeth [6]. Complex healing processes take place to repair or regenerate damaged structures, but pulp necrosis or destruction of periodontal tissue architecture may occur. Whereas pulp necrosis can usually be treated without much difficulty, extensive damage to the periodontium is irrepairable. Thus, recent approaches in dental traumatology primarily explore anti-resorptive and regenerative therapies to preserve periodontal tissues [6]. Damage to pulp and periodontium may result in root resorption, a pathological process characterized by progressive loss of cementum and dentine due to clastic cellular activity [7]. Whereas resorptive processes in bone are mostly physiological as a constant turn-over and adaptive response to stress and stimulation [8], teeth are not subjected to physiological remodeling apart from resorption in deciduous teeth. Permanent teeth are protected from resorption by barriers, on the root surface by a layer of cementum, within the endodontium by a layer of predentine [9, 10]. Damage to these protective layers, e.g. after dental trauma, exposes the underlying dentine and makes it accessible to clastic cells [10], which can bind exclusively to mineralized tissue surfaces to initiate the resorptive process [11–13]. A recent investigation among adolescents reported a risk of root resorption after dental trauma to be 2.3% [14].

Different classifications of root resorptions can be found in the literature based on etiological aspects or stimulatory factors [15–18], but more commonly based on their anatomic location in relation to the root surface [19, 20]. The prognosis of teeth with root resorption is questionable, without therapeutic intervention poor. Root resorption after dental trauma in the permanent dentition remains a challenge to the dental practitioner, due to manifold etiology and diverse clinical appearance. To ensure prevention or early detection and optimal treatment, it is essential to understand the underlying pathophysiological processes. The aim of this scoping review was to extract all available information on the regulation and pathogenesis of root resorption from the literature, to identify knowledge gaps, to compile a concise overview and thus contribute to a better understanding of the disease patterns.

## Methods

### Protocol

The form of a scoping review was chosen as this type of review offers an innovative tool to delineate a broad research question in a systematic way and thus allows for an assessment of valid knowledge, knowledge gaps and the needs for future research [21]. The protocol was not prospectively published or registered with PROSPERO (International prospective register of systematic reviews) [22] as the focus of this study on basic science and the more explorative nature of this scoping review do not quite fit PROSPERO’s objectives aiming towards outcomes of direct patient or clinical relevance. In addition, PROSPERO denied registration of scoping reviews or literature scans at the time the study was conducted. This work was initially aligned to the applicable aspects of PRISMA [23]. As soon as the PRISMA extension for Scoping Reviews (PRISMA-ScR) became available, which better suited the needs for this study, the format was adapted [24]. A checklist of the preferred items according to PRISMA-ScR can be found in Additional file 1.

### Literature search and data management

The search included reports on clinical aspects and pathophysiological mechanisms of root resorption after dental trauma in the bibliographic databases listed in Table 1. The last search update was run on February 9th, 2021. The search strategies were based on the two concepts “dental trauma” and “root resorption” which were combined with the Boolean operator “AND”. No limits or search filters were applied. A medical librarian (HK) developed the search strategies in close cooperation with the domain experts. For each of the concepts, a broad range of synonyms and relevant subject terms from the databases’ thesauri was compiled. While the search strategy was not peer-reviewed, the study was conducted in adherence to the PRESS checklist [25]. The full electronic search strategies that allow for replication of searches can be found in Additional file 2.

**Table 1:** Data bases for electronic literature search

Name of database	Provider/interface	Time period covered
Embase	Ovid	1974–2021
MEDLINE	Ovid	1950–2021
*Cochrane Library*		
Cochrane Database of Systematic Reviews (CDRS)	Wiley Online Library	1995–2021
Cochrane Central Register of Controlled Trials (CENTRAL)	Wiley Online Library	1948–2021
Database of Abstracts of Reviews of Effect (DARE)	Wiley Online Library	1995–2015
NHS Economic Evaluation Database	Wiley Online Library	1994–2015
Health Technology Assessment Database (HTA)	Wiley Online Library	1988–2015
Science Citation Index Expanded	Web of Science	1965–2021

The database searches were complemented by manual searches. To amplify the search, the reference lists of the books and articles considered as most relevant were screened. When reviewing other texts, the full texts of references considered as potentially relevant were checked, as well as articles specified as recommended reading. This was amended by ad hoc searches in Google Scholar for specific aspects. Records from the searches were transferred to a reference manager software (Citavi 6, Swiss Academic Software GmbH, Wädenswil, Switzerland) for deduplication, title/abstract screening and subsequent study selection based on the full text.

### Eligibility criteria

While we employed no language filter in the database searches, manuscripts selected for review were limited to English and German language in order to cope with the large number of results (Table 2). Papers unrelated to pathophysiology of resorption in bone and teeth, unrelated to dental traumatology or related to implant prosthetics or veterinary medicine were excluded. Orthodontically induced root resorptions were not excluded a priori but excluded if unrelated to pathophysiological mechanisms. Clinical cases of dental trauma unrelated to root resorption were excluded as well as papers that clearly were no longer up to date, had been published repeatedly or had successors. Included were papers containing physiologic, histologic, anatomic and clinical aspects of root resorption as well as publications that were not primarily focused on trauma-induced root resorption but reported detailed insights into resorptive processes in general. Thus, numerous studies on orthodontically induced root resorption or resorption in deciduous teeth were included, along with material on the biology, physiology and histology of bone.

**Table 2:** Eligibility criteria

Inclusion	Exclusion
English and German language	No relation to pathophysiology of resorption in bone and teeth or dental traumatology
Content of physiologic, histologic, anatomic and clinical aspects of root resorption	Relation to implant prosthetics or veterinary medicine
	Orthodontically induced root resorptions if no relation to pathophysiological mechanisms
	Clinical cases of dental trauma without relation to root resorption
	Papers published or updated multiple times

### Data extraction and qualitative synthesis

Information was extracted from the articles identified as relevant after thorough inspection and sorted into the following categories based on their primary content: (1) anatomy and histology, (2) regulation and pathogenesis and (3) etiology. During this process, the outline of this manuscript was refined and complemented. Controversial statements and unsettled issues were identified and are pointed out in the results section. Numerous publications appeared to be similar regarding content and referenced sources. In that case, findings were reported while citing only representative works. The results section comprises the information extracted from all included articles in a compacted summary.

## Results

**Fig. 1:**
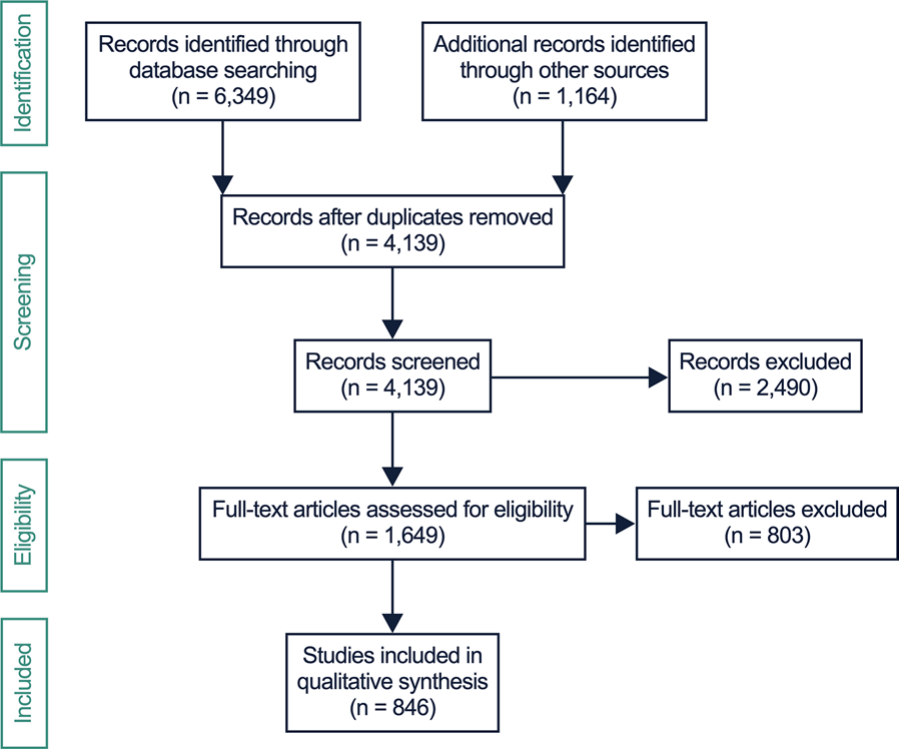
Information through different phases of the systematic scoping review based on the PRISMA guidelines [23]

The electronic and manual searches yielded 7513 records, 4139 remained after removal of duplicates to be subsequently screened for relevance on the basis of title and abstract. After screening, 2490 were excluded as non-relevant. The remaining 1649 full texts were reviewed for eligibility, and 803 were removed due to the exclusion criteria. Finally, 846 full text reports were summarized qualitatively. The process is illustrated in the PRISMA flowchart in Fig. 1 [23]. Many of the eligible articles contained identical or similar information in respect to the purpose of this study. Therefore, only representative sources were cited. Systematic reviews were found mainly on orthodontically-induced root resorption, reviews on trauma-induced resorption are scarce and do not focus on pathophysiological aspects.

### Physiological and pathological resorption in bone and the RANKL/RANK/OPG system

In bone, continuous remodeling enables functionality; it is essential during growth and for adaptation to ever-changing mechanical stresses [9, 26]. 10% of the total bone mass is exchanged annually, where bone remodeling prevents material fatigue and enables the repair of micro-trauma, along with the efficient mobilization of calcium [27]. This process is a quantitatively, spatially and temporally tightly regulated interplay of osteoclasts and osteoblasts [9, 11, 28]. Under physiological conditions, there is an equilibrium of bone formation and resorption, which is achieved through a finely tuned interaction of both processes called “coupling” [29]. A pathologic imbalance of this dynamic remodeling can lead to alterations of bone structure and stability [30].

The receptor-ligand system RANKL/RANK/OPG plays an essential role, these proteins of the TNF-family are key molecules regulating physiological and pathological resorption of mineralized tissues [31–34]. They control all aspects of osteoclast function [35, 36] and regulate the communication between bone cells and vascular as well as immune cells [37].

**Fig. 2:**
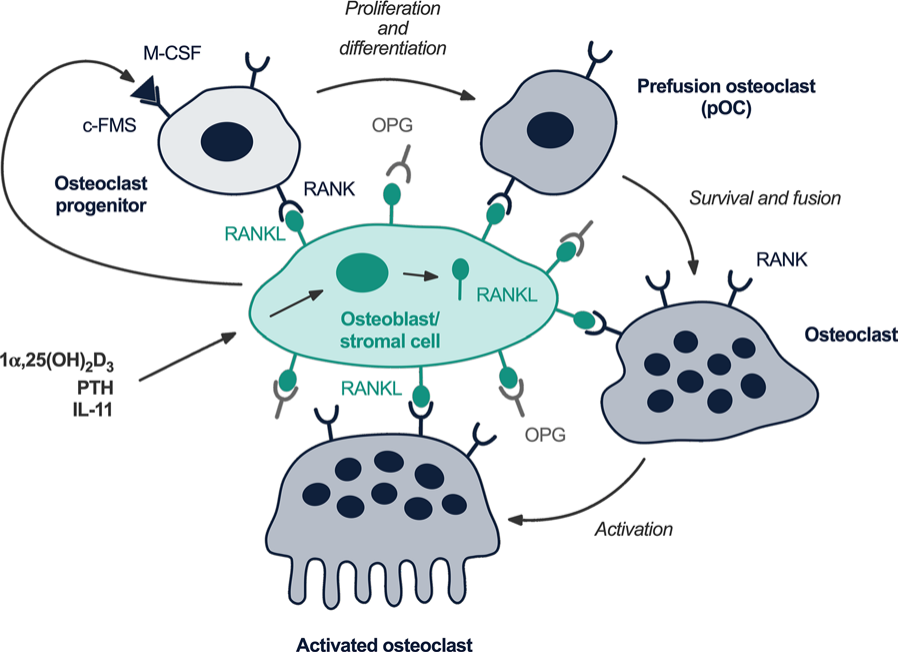
Schematic depiction of osteoclast differentiation and activation by osteoblasts/stromal cells [42]

RANKL is expressed by osteoblasts either as membrane-bound or soluble protein [37]. Osteoclasts, their mononuclear precursors as well as dendritic cells carry the respective receptor called RANK [38, 39]. Binding of RANKL to RANK starts signal transduction via the transcription factor NF-κB, induces fusion of osteoclast precursors into multi-nucleated cells and thus stimulates their maturation (Fig. 2) [40–43]. Soluble OPG competitively binds to RANKL and efficiently inhibits osteoclast formation and thus bone resorption [44]. Local and systemic factors influence this process [45]. Bone resorption and calcium release are stimulated by inflammatory cytokines or PTH, estrogen and calcitonin have the opposite effect [46, 47]. Progressive resorption due to elevated clastic activity is characteristic of diseases such as rheumatoid arthritis [48], osteoporosis [49] or periodontal disease [50]. Activation of immune cells leads to the release of cytokines like TNFα, which induces a complex inflammatory cascade characterized by increased cell differentiation, activation of osteoclasts and inhibition of apoptosis [34, 51, 52]. In periodontal disease, the cytokines IL-1, TNFα und IL-6 activate osteoclasts via RANKL, resulting in increased resorption of the alveolar bone.

### Etiological factors of root resorption

The development of any type of root resorption is tied to two premises: an initial injury and the subsequent persistence of a stimulus [15–17]. The injury damages the protective layer of root dentine [53]. Cementoblasts are destroyed directly or become necrotic as a result of a compromised blood supply to the periodontal ligament or dental pulp [54]. The lesion can be induced by trauma, surgical procedures or periodontal treatment [53], by pressure caused by impacted teeth, cysts or tumors [12, 55], or chemically by caustic compounds such as hydrogen peroxide used for internal bleaching [56]. Increased tissue pressure is also generated during orthodontic treatment, where complications such as apical root resorption can lead to significant root shortening [57]. Excessive occlusal load can promote resorption [58], but interestingly also the absence of physiological stresses in the case of orthodontic movement in non-occlusion [59]. Furthermore, infections of the root canal or periodontal ligament may trigger resorption [18].

Only a few systemic and endocrine diseases involve root resorption, which shows again that teeth provide a remarkable resistance to resorption. Links have been observed for hypo- as well as hyperparathyroidism, calcinosis, Gaucher’s disease, Turner syndrome, Paget’s disease and Herpes zoster [18]. Root resorption due to systemic disease can be found most commonly bilaterally and at the root apex. Resorptions are termed idiopathic if no local or systemic factors can be identified, however, there are only few reports in the literature. Idiopathic resorptions were found in single or multiple teeth, did not cause symptoms, and were incidental radiographic findings [60]. A cervical type progressing towards the pulp has been distinguished from an apical type, which progresses towards the coronal aspect of the tooth, causing successive root shortening [61].

### Types of root resorption

The type of resorption which develops depends on the type and intensity of the initial injury, the stage of root development and on the pulpal status [62]. Whereas the initial injury may be similar for different types of resorption, the subsequently dominating stimulus, which clastic cells depend on during phagocytosis [63], is decisive for further progression. Without a constant stimulus, the resorptive process is self-limiting and will arrest within 2–3 weeks [64]. This explains the phenomenon of transient resorption, which occurs to varying degree after minor traumatic impact and stops spontaneously without therapeutic intervention [15, 65]. Small and localized damage to root cementum can regenerate after a transient, self-limiting process, where neighboring intact cementoblasts repair the defect [15].

Severe damage to cementum affecting more than 20% of the root surface leads to extensive cell necrosis and will not heal spontaneously, resulting in replacement resorption [53, 66]. Loss of the protective barrier exposes dentine, which becomes part of the remodeling process in bone, leading to ankylosis of the respective tooth [67]. After severe traumatic impact, in particular after intrusion and avulsion, replacement resorption is likely to occur, with increasing risk with unfavorable storage of the tooth, particularly dry storage for more than 60 min [68]. Recent studies and a systematic review reported the development of replacement resorption in more than 50% of replanted teeth after avulsion [69, 70]. Transformation of root dentine into bone may be considered an “error” of the resorbing cells, which are unable to distinguish between these two types of tissues [15, 18]. Replacement resorption can be classified as a special type, as it progresses without additional stimulatory factors. It occurs after the acute inflammatory response has faded, cannot be associated with a bacterial stimulus and is resistant to therapeutic intervention [71, 72]. It advances to the point of complete replacement of the root by bone, leading to tooth fracture and loss of the crown [65]. The progression rate depends on the patient’s age and is faster in adolescents [73] compared to older patients. As today’s treatment for root resorption targets removal of the continuous stimulus, promising therapeutic concepts for replacement resorption have not been introduced, merely strategies to minimize the extent of damage [74].

External inflammatory resorption develops due to combined injuries to pulp and root cementum, particularly after luxation injuries [75, 76]. It is driven by intracanal infection after pulp necrosis in combination with damage to the cementum. Bacterial toxins, which penetrate through the dentinal tubules and beyond the damaged root surface, represent a strong stimulus to clastic cells and stimulate a rapidly progressing resorptive process [64]. The assessment of inflammatory mediators collected from gingival crevicular fluid in traumatized teeth showed a marked increase of IL-1α, Il-1β and TNFα in injured versus control teeth, and IL-1α was suggested as a potential biomarker for the early detection of external inflammatory root resorption after trauma [34]. This type of resorption is the result of a severe luxation and neglect of root canal treatment. After root canal disinfection, the resorptive process stops. Combinations of inflammatory and replacement resorptions are possible; in this case, root canal treatment can slow down the resorptive process considerably [1].

The etiology and pathophysiological mechanisms of cervical resorption is not yet fully understood. Triggering factors besides dental trauma may be bacteria from the gingival sulcus [17], anatomic irregularities of the enamel–cementum border or damage to the periodontal ligament by subgingival debridement [77–80], but also internal bleaching or previous orthodontic treatment [7, 15, 17, 81]. Heithersay claims that initial invasion of a fibrovascular tissue with a secondary bacterial trigger and not primary microbiological involvement is responsible [82]. Data from a larger clinical study indicated that in nearly two thirds of cases, more than one potentially predisposing factor was identified, thus cervical resorption may be multifactorial [83]. Recently, diabetes has been suggested as a predisposing systemic factor for cervical resorption, as inflammation and oxidative stress, the activation of clastic cells and a promotion of angiogenesis relate to the hypoxic cellular microenvironment in these patients [84]. This finding is supported by a case report and immunohistochemical analysis of a tooth with cervical resorption after extraction, which revealed an increase of hypoxia-inducible factor 1α-positive cells within the lesion; at the same time ectopic calcifications in the pulp confirmed the hypoxic environment [85].

Internal resorption occurs after loss of the protective predentine in combination with a continuous bacterial stimulation, where infected or necrotic pulp tissue coronally, but vital tissue apically of the resorptive process is imperative for progression [12, 18]. Bacteria may penetrate via dentinal tubules, carious lesions, along fracture lines or lateral canals. Since these premises are seldom met, internal resorptions are rare [15]. Whereas etiological factors are not certain, dental trauma is a likely cause [86], but periodontal disease, carious lesions, chronic pulpitis, vital pulp treatment or iatrogenic factors such as cavity preparation and orthodontic treatment have to be considered [12, 15]. Histologic analysis of teeth with internal root resorption reveal a multi-faceted character, a case report showed acute infection with multi-nucleated resorbing cells on the dentin surface at the resorption site, but normal tissue without inflammatory changes apically of the lesion in an upper canine, a second case was characterized by chronically inflamed granulation tissue with massive bacterial colonization [87].

The different type of root resorption, primary cause and additional stimuli are listed in Table 3.

**Table 3:** Classification of root resorption

	Primary cause	Additional stimuli
Transient resorption	Spatially limited damage to root cementum	
Replacement resorption	Massive damage to root cementum (> 20% of root surface)	
External inflammatory resorption	Massive damage to root cementum	Root canal infection
Cervical resorption	Damage to root cementum (?)	Hypoxic micro-environment (?)
		Spatially limited damage to periodontal tissues
		Compromised blood supply
		Sulcular infection (?)
		Non-overlapping enamel and root cementum with exposed dentin surface (?)
		Bleaching
		Orthodontic treatment (?)
		Periodontal treatment (?)
		Endocrine/autoimmune (?)
Internal resorption	Damage to predentine	Pulp infection coronally of the resorptive defect, apically vital tissue

### Protective mechanisms in permanent teeth

The dental hard tissues cementum, dentine and enamel are not resorbed under physiological conditions; even periradicular lesions cause the resorption of bone, but rarely of dentine. Different inhibitory measures have been discussed, among them remnants of HERS, which surrounds the tooth root like a net [9]. Whereas expression of amelogenin by remnant HERS cells may have inhibitory effects on a pathological destruction of cementum [88], the role of HERS as a protective mechanism remains open.

A key factor appears to be a protective lining of the root surfaces, on one hand by precementum, on the other hand by odontoblasts and predentine [13, 89]. Cervically, the root is covered by acellular cementum, the apical third contains cellular cementum with cementocytes in lacunae and a cover layer of cementoblasts, which form a non-mineralized cementoid [9]. Osteoclasts are not capable of binding to non-mineralized surfaces [17]. Damage to this protective layer leads to exposure of dentine, where osteoclasts bind to cell-adhesive peptide sequences (RGD: arginine-glycine-aspartic acid) of matrix proteins via their integrin receptors [90, 91]. Within the root, odontoblasts and predentine form a non-mineralized organic layer, which similarly inhibits resorption. In cervical resorption, a 200 µm resorption-resistant layer made of predentine, dentine and mineralized repair tissue around the root canal prohibits penetration of the resorptive process into the pulp space. This layer can typically be observed as a characteristic radiopaque sheath around the root canal [79, 80]. The PDL, which consists of collagenous fiber bundles bridging the gap between root cementum and alveolar bone also forms a barrier. PDL cells are involved in the formation and degradation of bone, cementum and collagen bundles [92]. They produce protease inhibitors, which prevent the adhesion of osteoclasts and the invasion of bone cells into the PDL compartment [93, 94].

Another protective barrier is the hyaline layer of Hopewell-Smith, a hypercalcified coating which aids cementum in binding to dentine. This layer prevents the penetration of bacterial products from the root canal into the PDL and vice versa [95]. Its destruction after traumatic impact in combination with a root canal infection stimulates clastic cells and leads to a rapidly progressing resorption. Further intrinsic factors of cementum and dentine can inhibit the formation and activity of clastic cells. Cementoblasts and also cementocytes, in comparison to the structurally similar osteoblasts and osteocytes, produce notably higher levels of OPG, where an increased ratio of OPG to RANKL constitutes another mechanism to prevent the resorption of cementum [96].

### Cells involved in root resorption

Resorption requires an elaborate interaction between resorptive osteoclasts or odontoclasts and immune cells such as monocytes, macrophages and dendritic cells [61, 97, 98].

Osteoclasts are multi-nucleated cells of 30–100 µm in diameter, which are capable of bone resorption [99] and play a central role both during physiological and pathological processes [100]. Clastic cells originate from monocytic hematopoietic progenitors of bone marrow [46, 47, 101, 102]. After their maturation, the process of subsequent fusion into multi-nucleated clastic cells is influenced by multiple cytokines and growth factors, particularly M-CSF and RANKL (Fig. 2) [42, 103]. M-CSF binding to its receptor induces proliferation of progenitor cells and expression of the receptor RANK [104], at the same time suppressing OPG [105]. In differentiated osteoclasts, M-CSF increases cell motility and protects them from apoptosis [106]. RANKL binding to RANK as well as M-CSF induce differentiation of osteoclasts. These are activated by RANKL and IL-1, become polarized and extend pseudopods and filopods, which enable ameboid movement [107]. They align along indentations called Howship lacunae, form a sealing zone which confines the resorptive area and disintegrate the mineralized tissue along protuberances called the “ruffled border” [108]. Whereas mononuclear osteoclasts are also capable of resorption, multi-nucleated cells are predominant as they appear to be more efficient, although cell fusion requires a high energy consumption [109, 110]. Osteoclasts resorb up to 100 µm^2^ of bone per day, which corresponds to the activity of 100 osteoblasts and reflects the high efficiency of these cells [111]. Clearance of degradation products is followed by apoptosis of osteoclasts or return to a non-resorbing state [108].

Similarly, odontoclasts are multi-nucleated cells that resorb dental hard tissues. They resemble osteoclasts and originate from the same progenitors. However, there is still no agreement as to whether odontoclasts and osteoclasts are the same [61, 112]. Odontoclasts exhibit equal ultrastructural and histochemical properties [113–115], resorb their substrate in the same manner [116], use the same key enzymes [117, 118], form Howship lacunae, appear polarized, form a ruffled border [116] and express RANK [38]. On the other hand, odontoclasts are smaller, have fewer nuclei, form smaller sealing zones [119–121], and the calcitonin-receptor has not been identified in these cells [118]. However, odontoclasts may form two ruffled borders and resorb dentine and bone at the same time [122], and osteoclasts have been described to be capable of dentine resorption [123, 124]. Therefore, it can be deducted that osteoclasts and odontoclasts are very similar.

Macrophages, monocytes and dendritic cells derive from the same hematopoietic progenitors as osteoclasts and play important roles in the resorptive process [96, 125, 126]. Monocytes are leucocytes, which circulate in blood but can migrate into adjacent tissues and transform into macrophages. Attracted by chemotactic signals, monocytes and macrophages take up and remove foreign matter and pathogens by means of cytoplasmic, acid-containing granula, which allow them to digest debris [61, 127]. Macrophages are structurally similar to osteoclasts and remove tissue remnants generated during the resorptive process [96, 128]. Dendritic cells have so far been looked upon as cells with solely immunologic function, but immature dendritic cells can also differentiate into clastic cells [129]. Their presence in dental pulp leads to the assumption that they are progenitors of odontoclasts [55].

### Pathogenesis of root resorption

To initiate the resorptive process, osteoclasts have to migrate, fuse, adhere to the surface, polarize and form new membrane domains, then demineralize hydroxyapatite and disintegrate the organic matrix. Immunological mechanisms play an essential role for the initiation and continuation of pathological resorption [130, 131]. Obviously, osteoclasts are the link between mineralized tissues and immune system, as they share the same progenitors as classic immune cells [109]. The initial immune response is linked to the subsequent resorption, where differentiation of clastic cells is part of the repair process. Traumatic injuries cause tissue damage; necrotic cells and local release of cytokines and chemokines attract T-cells, which in turn recruit and activate macrophages and granulocytes. Disintegration of tissue barriers allows microorganisms to enter, and bacterial toxins fortify this process. Macrophages ingest tissue debris and microbes, and produce cytokines such as IL-1β and TNFα, but also calcitriol, PGE2 and dexamethasone, which stimulate the expression of RANKL in PDL fibroblasts and T-cells [132, 133]. Several studies describe the regulation of bone resorption, but there is evidence that the same proinflammatory cytokines, namely IL-1 and TNFα are involved in root resorption [134, 135].

The process of cell adhesion appears to be indispensable for osteoclast maturation [136]. Binding to dentin sialoprotein and the RGD-motif [137] via the integrin receptor causes a reorganization of the cytoskeleton and the stimulation of resorption [138]. Integrins and cadherins furthermore enable cell mobility, signal transduction, matrix recognition and induction of resorption [108, 139]. Interestingly, clastic cells bind to dentine better than to bone, the sealing zone shows a longer half-life, and resorption lacunae develop faster [140, 141]. This characteristic structure drives the actual resorption, where hydroxyl- and chloride ions are pumped into the Howship-lacuna to form hydrochloric acid, the pH drops to 4.5 and hydroxyapatite crystals disintegrate [142, 143]. Subsequently, enzymes such as TRAP or cathepsin K, procollagenases and matrix metalloproteinases are secreted to degrade the organic matrix [46, 139]. In combination, acid and proteases successively degrade the mineralized tissue [144], and calcium and phosphate ions as well as collagen fragments are removed by the osteoclast via transcytosis. Whereas pathophysiological processes during bone resorption have been described, and attention has been paid to resorption after orthodontic movement or in deciduous teeth, studies on trauma-induced root resorption are rare. As evidence is accumulating that cells and mediators are identical, it appears likely that insights from research on bone is transferrable to the resorption of dentine and cementum during root resorption. RANKL/RANK/OPG control bone resorption as well as root resorption, and the underlying cellular mechanisms appear to be alike [105, 118, 132, 145–147]. RANKL is expressed by osteoblasts, but also odontoblasts, pulp- and PDL-fibroblasts, cementoblasts and activated T-cells [37], OPG by odontoblasts, ameloblasts, pulp- and PDL-fibroblasts [148, 149]. RANKL can be found in deciduous teeth undergoing resorption [118, 150, 151], on the contrary, PDL cells in non-resorbing deciduous teeth and in permanent teeth express OPG but not RANKL. Similarly, RANKL is involved in root resorption due to mechanical stress during orthodontic movement, where PDL cells increase RANKL, but decrease OPG expression [133, 145, 146, 152]. Evidence from the literature is sparse, but it seems that the underlying mechanisms of RANKL expression and OPG suppression are similar after dental trauma. Interestingly, topical treatment of the root surface with denosumab, a human monoclonal antibody that mimics the effects of osteoprotegerin in bone metabolism, inhibited the expression of RANKL and reduced root resorption after 2 months considerably in a rat model after extraction and replantation after an extraoral dry time of 60 min [153]. Topical application of alendronate, an osteoclast inhibitor used to treat osteoporosis, showed similar effects [154]. Along these lines, the application of the anti-oxidant N-acetylcyteine as intracanal medicament in a similar animal model led to reduced levels of RANK, the number of clastic cells and the extent of resorption [155]. Such findings highlight the potential of pharmacological approaches to decrease the extent of resorptive processes in situations which are currently not susceptible to therapeutic intervention. Bacterial infection often accompanies the progression of root resorption. PDL cells stimulated with *Prevotella intermedia* or *Porphyromonas gingivalis* increase RANKL-production [156]. LTA, the biologically active surface component of grampositive bacteria may cause resorption via the same mechanism [157].

Whereas in most resorptive processes, resorption appears to be dominant, cervical resorption shows different phases [158]. During the initiation stage, local destruction of the periodontium induces an immune response and formation of granulation tissue in contact with dentine, which can lead to clastic activity after activation of the RANK/RANKL system. The second phase of resorption is characterized by progression. A third, reparative stage can be observed later on, where formation of new bone-like tissue takes place. In cervical resorption, resorptive processes can be observed simultaneously with repair in different areas of the affected toot**h** [158]**.**

### Stimulators and inhibitors of resorption

Stimulators of bone resorption include systemic factors, such as PTH and calcitriol. PTH stimulates RANKL expression in osteoblasts and directly influences osteoclast fusion. Similarly, PDL cells react to PTH, which suggests that hormonal or mechanical stimulation may change the ratio of OPG and RANKL in these cells [132, 151, 159] and thus regulate the activity of periodontal tissues. Furthermore, PTH positively influences tooth eruption and orthodontic movement [160–162]. Calcitriol increases the resorptive activity of mature osteoclasts without increasing their quantity [61].

Besides systemic factors, locally expressed factors stimulate resorptive processes. Macrophages and leucocytes produce these cytokines during inflammation in response to bacteria, tissue debris and other cytokines, where most of these factors affect the RANKL/RANK/OPG system. IL-1 is involved in the resorption of periapical and periodontal tissues [163, 164], as it activates osteoclasts and stimulates the production of inflammatory mediators such as PGE2. IL-6 increases this effect by inducing RANKL expression in stromal cells and enhancing osteoclast differentiation [165]. PGE2 itself upregulates the activity of cementoclasts by controlling RANKL/OPG expression in cementoblasts [166], which illustrates the close interconnection of resorptive process and inflammatory response. Inflammation, vasoconstriction, enhanced clotting and transmission of pain are concomitant events of this process.

Bacteria themselves are stimulators of resorption, as they produce acids and proteases that degrade matrix components and tissues. During root resorption, LPS from cell walls of gramnegative bacteria stimulate osteoclast activity directly, but also indirectly by inducing the production of osteolytic factors by osteoblasts and macrophages. Enzymes and collagenases as well as IL-1, IL-6, M-CSF und PGE2 increase osteoclast activity [165]. It has been shown that bacteria such as *Treponema denticola, Porphyromonas gingivalis* and *Treponema socranskii* induce osteoclast formation via increased RANKL and PGE2 and decreased OPG [167]. Similar mechanisms have been observed for surface components of grampositive bacteria.

Inhibitory factors of clastic cells and thus resorptive processes include the hormone calcitonin, which reduces the motility of clastic cells and makes them withdraw from the resorption front. Calcitonin is effective at minute concentrations, its receptor CTR is expressed by osteoclasts [168, 169]. Inhibitory effects of calcitonin have also been demonstrated for odontoclasts, although the presence of CTR on these cells has not been proven yet [118, 170]. Inhibitory effects have also been shown for estrogen, furthermore for interferon and corticosteroids. Besides these systemic factors, again local cytokines inhibit clastic cells, among them IL-4, IL-8, IL-10 and IL-18 [171, 172].

FGF-2 may take on different roles, it appears to have stimulatory effects on osteoclast differentiation and increases PGE2 production [173], on the other hand it may antagonize M-CSF and directly inhibit osteoclast precursors [174].

## Discussion

In this scoping review, the existing knowledge of pathophysiological mechanisms of root resorption after dentoalveolar trauma was systematically searched, collected, processed, integrated and summarized in a condensed yet comprehensive form.

Standards exist for the methodological approach for systematic reviews and meta-analyses. For new forms of preparation of evidence such as scoping reviews or evidence maps, full consensus has yet to be achieved. The existing literature exhibits discrepancies in recommended methods, and consistent definitions are lacking. Recent methodological papers present specifications regarding the approach and nomenclature and confine scoping reviews from the classical systematic reviews [68, 175–177]. Thus, the PRISMA-statement for systematic reviews and meta-analyses was adapted to the PRISMA-ScR checklist, which provides guidance [24]. The scoping review differs from a systematic review by asking a broad question instead of posing a precise problem and narrow topic. For systematic reviews, the literature search follows strictly defined inclusion- and exclusion criteria, oftentimes the choice of publications is limited to a specific study design [175]. Systematic reviews are based on the PICO scheme explicitly following the parameters patient/population, intervention, control intervention and outcome. The design may prohibit a comprehensive and yet up-to-date overview of the topic of choice, including all facets and sub-categories. Therefore, there is an increasing demand for different forms, which systematically summarize recent research findings and present evidence as well as evidence gaps [175]. Increasing numbers of scoping reviews and evidence maps, which reflect these considerations, are published to date. Whereas scoping reviews and evidence maps assess where consolidated knowledge ends and additional research is needed, systematic reviews clarify whether existing knowledge is reliable. Differences between the two forms of review are summarized in Table 4 [175]. With regard to the topic and aim of this work, a scoping review appears to be the ideal format [178]. Although the search for our study might not be as sensitive as for a systematic review, the literature identified to answer the question for this review was exhaustive. Since information on the regulation of trauma-associated root resorption is scarce, a strength of this review may be the synthesis of research findings from different fields, as knowledge, e.g. from mineralized tissue research on pathological resorption as well as from orthodontics is transferrable and applicable. Thus, a more detailed picture of root resorption can be presented.

**Table 4:** Comparison of systematic and scoping reviews [164]

	Systematic review	Scoping review
Aims	Systematic assessment and evaluation of evidence	Systematic mapping of evidence and identification of gaps
		Evaluation whether systematic review is necessary
		Identification of research needs
Question	Precise, narrow	Broad, beyond interventions, disease patterns or diagnostic measures
	Similar to primary studies	
		No limitations in regard to the question, control interventions or command variable
Study Design	Determined by question	Any
Quality Assessment	Yes	Not intended
Presentation of Results	Descriptive, often quantitative	Descriptive
		Unweighted evidence

A disadvantage of the chosen research concept is the large number of records from the database searches which needed intellectual selection imposing a substantial amount of effort. Inclusion and exclusion criteria for the present study were not as strictly defined as it is typical for systematic reviews, therefore the material was selected by thematic content rather than set characteristics of study design and factual accomplishment. The selection process required an individual, topic-based decision of whether to include or exclude each of the articles found after the primary search. This individual interpretation and rating of relevance influences the selection and eventually the body of literature that the analysis and summary of factors was based on, which might have resulted in bias and a contortion of the presented results. However, sensitive searching of multiple databases in combination with the manual searches and the resurvey of the relevant literature based on the demarcation set for this study counteracts this possible source of bias.

The thorough search and intense review of the existing literature revealed a body of knowledge, but also redundant information, controversial findings, knowledge gaps, lack of evidence and the need for further research. Repeated speculation without concrete sources can be found, which suggests that many aspects of this topic have not been explored and understood. This may be due to the complexity of regulatory processes, minor awareness of the problem, and a lack of suitable research models. Subsequently, late detection of root resorption is still common, which can result even in the loss of teeth, often in a critical zone of growth and esthetics, with rather unfavorable long-term consequences for patients which could have been prevented otherwise. For clinical practice, the understanding of etiological factors and the distinction of whether or not they can be influenced implies therapeutic approaches. Thus, more detailed insight into the pathomechanisms of cervical resorption might lead to more effective prevention and treatment. Replacement resorption poses another great challenge: whereas antiresorptive application of tetracycline [179] and/or corticosteroids [179] may inhibit clastic cells and thus defer and limit the extent of resorption, the process is unstoppable once it has commenced. Future research towards local pharmacologic approaches to prevent remodeling of dentine into bone harbors potential for major benefits for trauma patients. The establishment of animal models to study dentine resorption, e.g. by ectopic implantation of dentine chips after different pre-treatment may help to test the influence of different parameters on the resorptive process and develop strategies to control or avert resorption. Last but not least, more systematic education and thorough training of dental students and young professionals will be an important step towards prevention, early detection and optimized therapy of root resorption in patients who have suffered from dental trauma.

## Conclusion

The comprehensive overview of the pathophysiological mechanisms of trauma-induced root resorption may contribute to an increase awareness, draw attention to this complex topic and generate future research activities. This may impact patient care and improve the long-term prognosis of teeth that are at the risk of root resorption.

## Supplementary Information

**Additional file 1: **Preferred reporting items for systematic reviews and meta-analyses extension for scoping reviews (PRISMA-ScR) checklist.

**Additional file 2:** Electronic Search Strategies as exported from the search interfaces with only some formatting applied.

## Data Availability

The dataset used and/or analyzed during the current study available from the corresponding author on reasonable request.

## Abbreviations

PRISMA(-ScR): Preferred reporting items for systematic reviews and meta-analysis (extension for scoping reviews)
RANKL: Receptor activator of NF-κB ligand
RANK: Receptor activator of NF-κB
OPG: Osteoprotegerin
PTH: Parathyroid hormone
HERS: Hertwig’s epithelial root sheet
PDL: Periodontal ligament
M-CSF: Macrophage colony-stimulating factor
PGE2: Prostaglandin E2
LTA: Lipoteichoic acid
LPS: Lipopolysaccharide
FGF-2: Fibroblast-growth-factor 2
