# Supplementary material for: Pathophysiological mechanisms of root resorption after dental trauma: a systematic scoping review
Source: BMC Oral Health. 2021 Mar 26;21:163. doi: 10.1186/s12903-021-01510-6 (PMC7995728; doi:10.1186/s12903-021-01510-6)
Supplement: Supplementary file 2 — Additional file 2: Electronic Search Strategies as exported from the search interfaces with only some formatting applied. [file 12903_2021_1510_MOESM2_ESM.docx]

# Additional File 2: Electronic Search Strategies as exported from the search interfaces with only some formatting applied.

## Embase

**Host:** Ovid

**Database segments:** Embase 1974 to 2021 February 08 (oemezd)

**Date of search:** 2021/02/09

| **Set** | **Query** | **Records** |
| --- | --- | --- |
| 1 | (((tooth or teeth or dens or dentes or canin$ or incisor$ or incisivi or cuspid$ or bicuspid or premolar$ or molar$ or dental or cervix or cervic$ or root$ or cement$) adj6 (resorpt$ or resorb$)) or ((EARR or ERR) and tooth)).ti,ab. or ((exp cytokine/ or cytokine$.ti,ab.) and (resorpt$ or resorb$).ti,ab.) or (odontoclast$ or cementoclast$).ti,ab. or (osteoclast/ or osteoclast activity/ or osteoclastogenesis/ or osteoprotegerin/ or osteoclast differentiation factor/) or (osteoclast$ or Osteoprotegerin or RANK Ligand or RANKL or Receptor Activator of Nuclear Factor kappa B).ti,ab. or osteolysis/ or (osteoly$ or ((bone$ or alveol$) adj3 (loss$ or resorpt$ or resorb$ or atroph$))).ti,ab. | 155,777 |
| 2 | exp tooth injury/ or tooth replantation/ or ((tooth or teeth or dens or dentes or canin$ or incisor$ or incisivi or cuspid$ or bicuspid or premolar$ or molar$ or dental) adj6 (luxat$ or subluxat$ or contus$ or dislocat$ or exarticulat$ or intrus$ or extrus$ or avuls$ or ankylos$ or trauma$ or injur$ or fractur$ or replant$ or reimplant$ or re-implant$ or auto transplant$ or autotransplant$)).ti,ab. or ((root or dentoalveol$ or alveol$ or periodontal ligament$ or periodontium or periodontal pocket$) adj6 (contus$ or ankylos$ or trauma$ or injur$ or fractur$)).ti,ab. | 31,166 |
| 3 | 1 and 2 | 2,229 |

## MEDLINE

**Host:** Ovid

**Database segments:** Ovid MEDLINE(R) ALL 1946 to February 08, 2021 (medall)

**Date of search:** 2021/02/09

| **Set** | **Query** | **Records** |
| --- | --- | --- |
| 1 | exp tooth resorption/ or ((tooth or teeth or dens or dentes or canin$ or incisor$ or incisivi or cuspid$ or bicuspid or premolar$ or molar$ or dental or cervix or cervic$ or root$ or cement$) adj6 (resorpt$ or resorb$)).ti,ab. or ((EARR or ERR) and tooth).ti,ab. or ((exp cytokines/ or cytokine$.ti,ab.) and (resorpt$ or resorb$).ti,ab.) or (odontoclast$ or cementoclast$).ti,ab. or (osteoclasts/ or Osteoprotegerin/ or RANK Ligand/) or (osteoclast$ or Osteoprotegerin or RANK Ligand or RANKL or Receptor Activator of Nuclear Factor kappa B).ti,ab. or osteolysis/ or (osteoly$ or ((bone$ or alveol$) adj3 (loss$ or resorpt$ or resorb$ or atroph$))).ti,ab. | 106,146 |
| 2 | exp tooth injuries/ or tooth replantation/ or ((tooth or teeth or dens or dentes or canin$ or incisor$ or incisivi or cuspid$ or bicuspid or premolar$ or molar$ or dental) adj6 (luxat$ or subluxat$ or contus$ or dislocat$ or exarticulat$ or intrus$ or extrus$ or avuls$ or ankylos$ or trauma$ or injur$ or fractur$ or replant$ or reimplant$ or re-implant$ or auto transplant$ or autotransplant$)).ti,ab. or ((root or dentoalveol$ or alveol$ or periodontal ligament$ or periodontium or periodontal pocket$) adj6 (contus$ or ankylos$ or trauma$ or injur$ or fractur$)).ti,ab. | 29,439 |
| 3 | 1 and 2 | 2,421 |

## Cochrane Library

**Host:** Wiley Online Library (https://www.cochranelibrary.com)

**Databases:** CDRS (Cochrane Database of Systematic Reviews), CENTRAL (Cochrane Central Register of Controlled Trials)

**Date of search:** 2021/02/09

**Records found:** CDRS (3), CENTRAL (129)

| **ID** | **Search** | **Hits** |
| --- | --- | --- |
| #1 | [mh "tooth resorption"] or ((tooth or teeth or dens or dentes or canin* or incisor* or incisivi or cuspid* or bicuspid or premolar* or molar* or dental or cervix or cervic* or root* or cement*) near/6 (resorpt* or resorb*)):ti,ab or ((EARR or ERR) and tooth):ti,ab or (([mh cytokines] or cytokine*:ti,ab) and (resorpt* or resorb*):ti,ab) or (odontoclast* or cementoclast*):ti,ab or ([mh ^osteoclasts] or [mh ^Osteoprotegerin] or [mh ^"RANK Ligand"]) or (osteoclast* or osteoprotegerin or "rank ligand" or rankl or "receptor activator of nuclear factor kappa b"):ti,ab or [mh ^osteolysis] or (osteoly* or ((bone* or alveol*) near/3 (loss* or resorpt* or resorb* or atroph*))):ti,ab | 8,716 |
| #2 | ([mh "tooth injuries"] or [mh "tooth replantation"]) or ((tooth or teeth or dens or dentes or canin* or incisor* or incisivi or cuspid* or bicuspid or premolar* or molar* or dental) near/6 (luxat* or subluxat* or contus* or dislocat* or exarticulat* or intrus* or extrus* or avuls* or ankylos* or trauma* or injur* or fractur* or replant* or reimplant* or "re-implant*" or "auto transplant*" or autotransplant*)):ti,ab or ((root or dentoalveol* or alveol* or "periodontal ligament*" or periodontium or "periodontal pocket*") near/6 (contus* or ankylos* or trauma* or injur* or fractur*)):ti,ab | 1,475 |
| #3 | #1 and #2 | 132 |

## Science Citation Index Expanded

**Provider:** Web of Science

**Database segments:** Science Citation Index Expanded (SCI-EXPANDED), 1965-present; Data last updated: 2021/02/08

**Date of search:** 2021/02/09


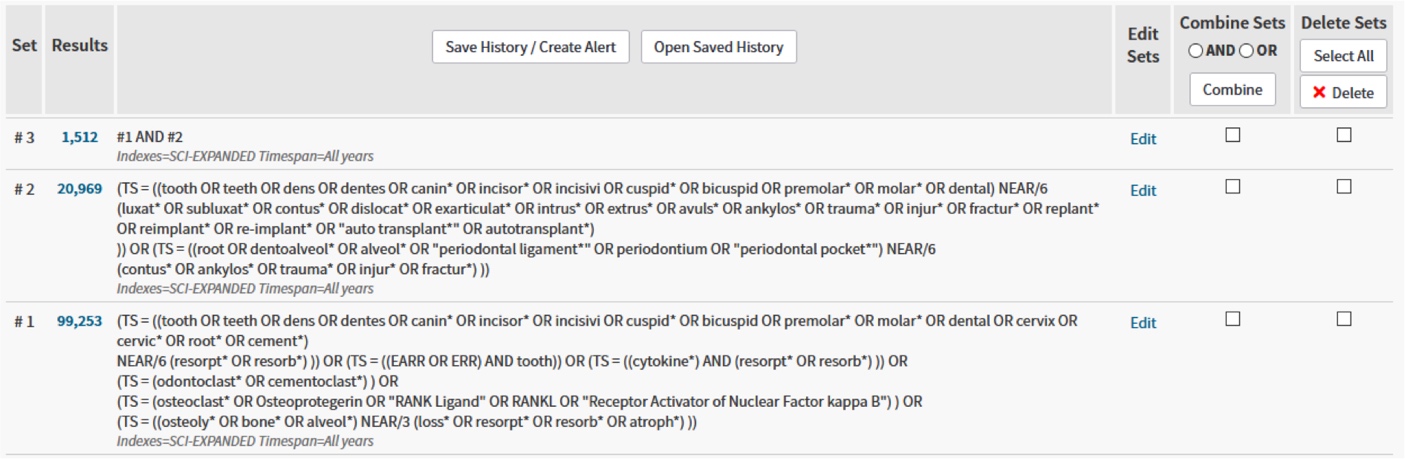


Web of Science does not provide a means to export the search strategy in text format. Therefore, in addition to the screenshot from the above web browser session we document the search strings as entered into the search form (advanced search). We hope this will be helpful when reproducing the search:

(TS = ((tooth OR teeth OR dens OR dentes OR canin* OR incisor* OR

incisivi OR cuspid* OR bicuspid OR premolar* OR molar* OR dental OR

cervix OR cervic* OR root* OR cement*) NEAR/6 (resorpt* OR

resorb*))) OR

(TS = ((EARR OR ERR) AND tooth)) OR

(TS = ((cytokine*) AND (resorpt* OR resorb*))) OR

(TS = (odontoclast* OR cementoclast*)) OR

(TS = (osteoclast* OR Osteoprotegerin OR "RANK Ligand" OR RANKL OR

"Receptor Activator of Nuclear Factor kappa B")) OR

(TS = ((osteoly* OR bone* OR alveol*) NEAR/3 (loss* OR resorpt* OR

resorb* OR atroph*)))

(TS = ((tooth OR teeth OR dens OR dentes OR canin* OR incisor* OR

incisivi OR cuspid* OR bicuspid OR premolar* OR molar* OR dental)

NEAR/6 (luxat* OR subluxat* OR contus* OR dislocat* OR exarticulat*

OR intrus* OR extrus* OR avuls* OR ankylos* OR trauma* OR injur* OR

fractur* OR replant* OR reimplant* OR re-implant* OR "auto

transplant*" OR autotransplant*))) OR

(TS = ((root OR dentoalveol* OR alveol* OR "periodontal ligament*"

OR periodontium OR "periodontal pocket*") NEAR/6 (contus* OR

ankylos* OR trauma* OR injur* OR fractur*)))

#1 AND #2
